# Interest in Infectious Diseases specialty among Japanese medical students amidst the COVID-19 pandemic: A web-based, cross-sectional study

**DOI:** 10.1371/journal.pone.0267587

**Published:** 2022-04-21

**Authors:** Hideharu Hagiya, Yuki Otsuka, Kazuki Tokumasu, Hiroyuki Honda, Yoshito Nishimura, Mikako Obika, Fumio Otsuka

**Affiliations:** Department of General Medicine, Okayama University Graduate School of Medicine, Dentistry and Pharmaceutical Sciences, Okayama, Japan; Gulu University, UGANDA

## Abstract

**Introduction:**

The emergence of the novel coronavirus disease of 2019 (COVID-19) has led to huge disruptions in the medical field and society. The significance of training and education for experts has been increasingly acknowledged in Japan, where the number of infectious disease (ID) specialists is reportedly insufficient. In this paper, we report the results of a web-based survey that was conducted to reveal the ways in which the COVID-19 pandemic has influenced medical students’ awareness of ID specialists and future career choices.

**Method:**

This cross-sectional descriptive study was conducted in March 2021 and targeted 717 medical students belonging to Medical School of Okayama University, Japan. The questionnaire consisted of four questions meant to assess students’ knowledge and future intentions of becoming ID specialists.

**Results:**

We obtained 328 eligible questionnaires (response rate: 45.7%). Of 227 (69.2%) students who were aware of ID specialists, 99 (43.6%) answered that they came to know about them only after the pandemic, 12 (3.7%) answered that their interest in being an ID specialist arose during the pandemic, while 36 (11.0%) responded that they would rather not become ID specialists. At the time of the survey, 5 students (1.5%) were aiming to become ID specialists.

**Conclusion:**

We observed a very low rate of interest to be an ID specialist among medical students. The experience of the pandemic does not seem to have influenced Japanese medical students to choose ID as a specialty for their careers. Continuous efforts to increase the number of ID specialists are necessary in Japan as a countermeasure against the coming pandemic.

## Introduction

The novel coronavirus disease of 2019 (COVID-19), originally reported in Wuhan, China, developed a global pandemic [1]. Despite the international efforts to highlight the importance of infection control and prevention against SARS-CoV-2, the unprecedented virus has repeatedly caused surges and outbreaks everywhere in the world [2,3]. To appropriately respond to the spread of unknown viruses, every healthcare facility has faced with extensive needs for ever-changing burdens. Repetitive emergences of the genetic variants have made things more complicated, urging us to manage this infectious disaster in a rapid and flexible manner [4,5]. With expert knowledge and experiences in infection control and prevention, as well as diagnosis and treatment, the infectious disease (ID) physicians have been expected to demonstrate clinical and administrative leadership. In fact, amid the two years of this pandemic, majority of them have contributed to directly managing patient care and organizing an infection control team by healthcare facility level and/or administrative level.

In this ongoing global pandemic, the need for human resource development in the ID field has been increasingly acknowledged. Currently, the number of ID specialists in Japan is insufficient, with the Japanese Society of Infectious Diseases reporting that there were 1691 certified specialists as of 15^th^ March 2022 [6]. A shortage of ID specialists is also seen in the United States, where 41 out of 165 residency programs in 2021 were reportedly unfilled [7]. The necessity of ID specialists is thus noted not only for the fight against COVID-19 but also for combating the global issue of antimicrobial resistance (AMR) organisms from a long-term perspective. We suspect that a principal cause for this shortage is the lack of awareness of ID specialists among medical students. We conducted a survey on how the COVID-19 pandemic affected Japanese medical students’ attitudes and future career choices.

## Materials and methods

In March 2021, a cross-sectional descriptive epidemiological study was conducted on all the medical students belonging to Medical School of Okayama University (Japan), using a web-based questionnaire with Google Forms. Informed consent was obtained from each student when answering the questionnaire. The review board of Okayama University Hospital approved the study (No. 2103–042). No particular exclusion criteria was established, and an access URL was sent to all eligible medical students in years 1–6 via email stating the purpose of the study. Responses were collected anonymously, and each student could answer only once. To increase the collection rate, the respondents were told that ten people would be selected by lottery to win a medical book of ¥5,000.

The questions in the web survey included the following items—(i) Have you heard about “ID specialists”? (ii) (If yes) Did you know about the existence of “ID specialists” before the COVID-19 pandemic? (iii) In the past, have you ever aimed to become an ID specialist? (iv) After experiencing the COVID-19 pandemic for a year, are you interested in becoming an ID specialist in the future?

Categorical variables were shown in numbers, percentages, and odds ratios (OR) with their 95% confidential intervals (CIs), which were assessed with the Chi-square test. All estimates were expressed as point estimates with 95% CI, and all reported *p*-values less than 0.05 were considered statistically significant.

## Results

Of 717 medical students, 329 (45.9%) students responded; however, one student did not agree to be included in the analysis, and thus, the final number of eligible answers for analysis was 328 (45.7% response rate) (212 males, 113 females, 3 did not specify gender) (**Table 1**).

**Table 1:** Numbers and proportions of answers obtained from the medical students.

	Year 1	Year 2	Year 3	Year 4	Year 5	Year 6	Total
Number of total students	113	119	123	126	124	112	717
Number (%) of answer	55 (48.7%)	44 (37.0%)	60 (48.8%)	62 (49.2%)	68 (54.8%)	39 (34.8%)	328 (45.7%)

### Awareness of ID specialists (Table 2)

In all, 227 (69.2%) medical students answered that they had heard about ID specialists. The awareness about ID specialists among younger grades before clinical clerkship was about half of the total answers (56.4% in Year 1, 47.7% in Year 2, and 56.7% in Year 3). On the other hand, it was higher among the students of Years 4–6 who had experienced clinical clerkship (54.1% vs. 83.4%; *p* <0.001; OR [95% CI], 4.3 [2.5 to 7.4]), with 74.2% in Year 4, 91.2% in Year 5, and 84.6% in Year 6. A total of 128 students (56.4%) knew the existence of ID specialists since before the COVID-19 pandemic. Comparing the younger and senior grade students, those during/after clinical clerkship knew the existence of ID specialists with a higher proportion (19.5% vs. 57.4%; *p* <0.001; OR [95% CI], 5.5 [3.3 to 9.5]).

**Table 2:** Questionnaire for awareness for Infectious Disease (ID) specialist among medical students.

	Before clinical clerkship			During/after clinical clerkship			Total	*p* values odds ratio (95% CI)
	Year 1	Year 2	Year 3	Year 4	Year 5	Year 6		
Have you heard about “ID specialists”?								
YES	31 (56.4%)	21 (47.7%)	34 (56.7%)	46 (74.2%)	62 (91.2%)	33 (84.6%)	227 (69.2%)	-
	86/159 (54.1%)			141/169 (83.4%)			-	*p* <0.001 4.3 (2.5 to 7.4)
Did you know about the existence of “ID specialists” before the COVID-19 pandemic?								
YES	7 (22.6%)	10 (47.6%)	14 (41.2%)	22 (47.8%)	46 (74.2%)	29 (87.9%)	128 (56.4%)	-
	31/159 (19.5%)			97/169 (57.4%)			-	*p* <0.001 5.5 (3.3 to 9.5)

COVID-19, coronavirus disease 2019; CI, confidential interval. P values were calculated by chi-square test.

### Past, current, and future intentions of becoming ID specialists (Fig 1)

When asked if they had ever thought of becoming an ID specialist, all students from Years 1 and 2 answered “Never,” indicating that none of them had any awareness of ID specialists at the time of admission. In total, 6.1% (20/328) responded that they had been interested in becoming ID specialists in the past but were not at the time of the survey. Overall, 5 out of 328 (1.5%) medical students, being equivalent to one student per Year, were aiming to become ID specialists.

**Fig 1:**
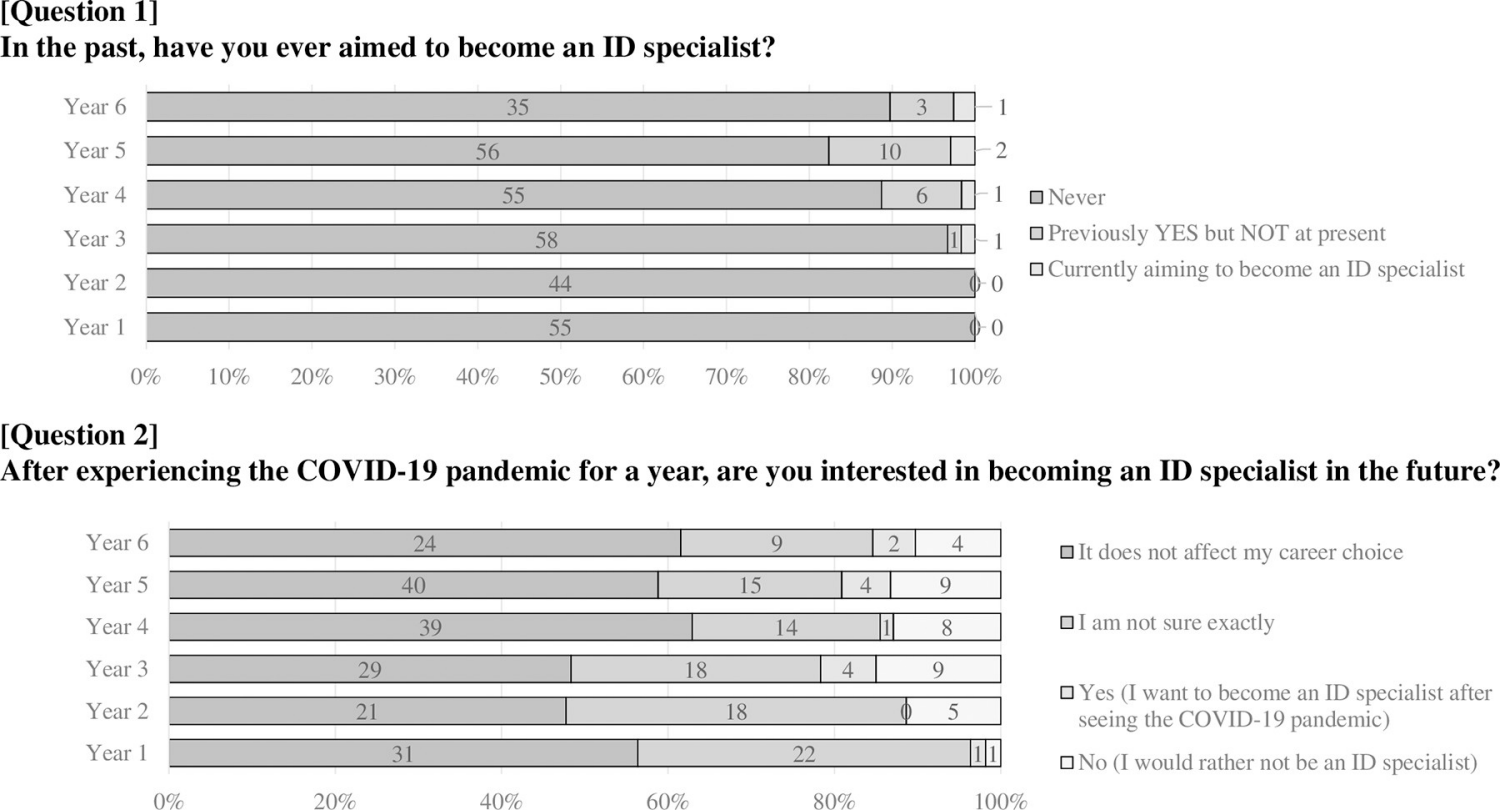
Questionnaire for past, current, and future hope for Infectious Disease (ID) specialists among medical students.

When asked if the experience of the COVID-19 pandemic influenced their career, approximately half the students in each grade answered that it had no bearing on their careers; nearly 20%–40% of the students were not sure about it. In all, 12 (3.7%) answered that their interest in being an ID specialist arose during the pandemic; however, 36 (11.0%) said they would rather not become ID specialists.

## Discussion

Through this survey, we found that medical students were not very keen on becoming ID specialists after one year of the COVID-19 pandemic. As of 31 January 2021, the number of members in the Japanese Society of Internal Medicine was 116,404 [8]; therefore, the proportion of ID specialists among internists was approximately 1.4% (the number of certified ID specialists was 1,622) [6]. This is equivalent to those who expressed their wish to become ID specialists in our study (1.5%; 5/328 students). We expected that the number of students pursuing ID specialties would increase after experiencing the pandemic; however, nearly half of the students responded that it would not affect their career choice, and, notably, 11% of the students had a negative preference for ID specialists.

Looking back, emerging pandemics such as Severe Acute Respiratory Syndrome (SARS) [9–11], the 2009 H1N1 influenza [12], and the Middle East Respiratory Syndrome [13] have significantly impacted medical students. The negative effects of the COVID-19 pandemic on medical education have also been reported in various fields such as surgical training [14], pre-clerkship, and clerkship learning [15,16], A survey on medical students in the United States suggested that about one-fifth of the respondents considered that the COVID-19 pandemic would unfavourably influence their choices of professional specialty [17]. Another report revealed that career choices for being good physicians markedly increased among two-thirds of students, although they focused on paediatric fields [18]; however, to the best of our knowledge, no survey has specifically investigated medical students’ career choices toward becoming ID specialists during an emerging pandemic.

Why did the COVID-19 pandemic not augment the number of medical students who aim to become ID specialists? We imagine that the anxiety against invisible fear might have prevailed over a sense of mission. According to a study conducted during the SARS crisis, medical students in Malaysia, especially those from the lower years, expressed a considerable sense of unease when going into hospital wards and observing or examining coughing patients [19]. Through daily contact with students, we feel that presently, newly admitted students tend to choose safer and easier careers in Japan. Thus, the fact that 11% of the students in our study had a negative impression of becoming ID specialists was sort of understand. The shortage of a role model for medical students could also be a discouraging factor, as the results in ID specialists’ career paths being even less visible. The presence of and exposure to role models in medical schools can greatly influence medical students’ choice of residency training and selection of future expertise [20,21]. In contrast to Japan and the United States [7], ID would be a popular specialty in other countries. For instance, ID was recently the most popular specialty among French medical students who were motivated by several factors such as a multi-system approach, experiences of an internship during medical school, the curable diseases by appropriate diagnosis and treatment, and its global appeal [22]. Further research is warranted to discover the underlying rationale in avoiding a major in ID as a future career option, which would help facilitate the career development of ID specialists. Additionally, Japanese national policies should address the uneven distribution of doctors by their specialties rather than its geographic unbalance [23].

The limitations of this study should be mentioned. First, it was difficult to draw solid conclusions owing to the lack of data before the pandemic. Second, since this study was conducted on medical students from a Japanese university alone, its results cannot be generalised. Third, the survey response rate was less than half (45.7%), and selection bias potentially exists. Forth, as the questionnaire was self-reported, information bias should also be considered. Assuming that non-responding students were not interested in the contents of the survey, we presume that the negative preference for ID specialists would be even greater than this result.

In summary, our data indicate that the unprecedented pandemic experience has not encouraged Japanese medical students to choose ID for their future specialty. However, if and when a new pandemic occurs in future, ID specialists are expected to play a leading role in implementing infection prevention measures in medical facilities and public health. Hopefully, even though it is a small number (12 students, 3.7%), our data suggest that some students became interested in becoming ID specialists during the pandemic. We need to continue our recruitment efforts to increase the number of ID specialists in Japan.

## Supporting information

S1 Table Raw data from the web-based questionnaire. (XLSX)
